# Environmental changes and their effects on carbon isotope distribution in Lake Plateliai over the past 130 years: insights from sediment organic fractions and diatom assemblages

**DOI:** 10.1371/journal.pone.0343824

**Published:** 2026-03-04

**Authors:** Rūta Barisevičiūtė, Jūratė Karosienė, Jūratė Kasperovičienė, Ričardas Paškauskas, Olga Jefanova, Piotr Szwarczewski, Žilvinas Ežerinskis, Laurynas Butkus, Justina Šapolaitė, Jonas Mažeika

**Affiliations:** 1 State Research Institute Center for Physical Sciences and Technology, Vilnius, Lithuania; 2 State Scientific Research Institute Nature Research Centre, Vilnius, Lithuania; 3 University of Warsaw, Warsaw, Poland; University of Maryland Center for Environmental Science, UNITED STATES OF AMERICA

## Abstract

In this study, we investigated how anthropogenic activities have affected the carbon cycle and biological productivity in a mesotrophic lake over the past 130 years. We analysed the carbon isotope distribution in two organic sediment fractions: alkali-soluble and alkali-insoluble along with diatoms and the organic matter content in the sediments. Over the course of 130 years, the freshwater reservoir age (RA) in both organic sediment fractions in this ecosystem changed by 872.4 ± 80.2 y. In this lake ecosystem, the values of specific radiocarbon (^14^C) activity in both sediment fractions remained very similar during the periods when the water level was kept more or less constant (1885–1932 and after 1985) and varied in the range of 1 pMC. Any changes in the plankton community affecting the ratio of stable carbon isotopes in the alkali-soluble fraction had no effect on the redistribution of ^14^C between the two organic sediment fractions. The differences in the RA up to 352.7 ± 57.4 y observed between the fractions during the Second World War and between 1960 and 1976 were associated with changes in the water level and the input of allochthonous substances into the lake ecosystem.

## Introduction

Radiocarbon (^14^C) dating of lake sediments is widely used to estimate the so-called freshwater reservoir age (RA), i.e., the ^14^C age difference between the contemporaneous organisms from the terrestrial environment and organisms that derive their carbon from the lake environment [1,2]. However, lake sediments are a mixture of autochthonous and various age allochthonous carbon sources having distinct ^14^C specific activities. The freshwater RA depends on the catchment bedrock, the CO_2_ exchange rates between water and the atmosphere, which are influenced by the production and decomposition rates of organic carbon, the inflow and outflow of organic and inorganic mater, water residence time, water level fluctuations, climate change, other environmental factors affecting the lake catchment area, and the change in the sedimentation rate [2–11]. In addition, the lake water and consequently the sediments may have different specific ^14^C activities at different locations within a lake, due to the proximity to the river input and the depth of the area, which affects photosynthetic activity [11–14]. Any disturbance that affects the carbon exchange between water ecosystem, terrestrial environment and the atmosphere will affect the carbon isotope distribution in the lake ecosystem.

Stable carbon isotope ratio analysis in carbonates (with stable oxygen isotope measurements) and sedimentary organic matter (OM) content and sedimentation rate measurements, C/N analysis in sedimentary OM, as well as biological proxy analysis (photosynthetic pigments, pollen, and diatoms) have been used for the reconstruction of events related to any changes in the climate and status of the lake ecosystem [15–21]. In the last twenty years, publications have appeared, using ^14^C activity distribution measurements within sediment layers as a proxy for these studies [14,22–25]. Radiocarbon measurements were even used to trace seasonal changes in autochthonous origin carbon fluxes to the lake’s ecosystem and how these changes affect the diet of aquatic organisms [26,27].

Lake Plateliai is the largest lake in the northwestern region of Lithuania, situated within the historical and cultural region of Samogitia. It lies within the Samogitia National Park, a protected area known for its pristine natural landscapes. Due to the absence of agricultural activity within the park, the lake remains one of the cleanest in Lithuania, with exceptionally clear waters, a well-preserved ecosystem, and water quality that meets the good ecological status [28]. The lake itself is important as a *Chara*-dominated habitat (classified as habitat type E3140: Hard oligo-mesotrophic waters with benthic vegetation of *Chara* spp.) under the EU Habitats Directive. This habitat is considered a priority by the EU as it is crucial for biodiversity, water quality monitoring, and understating of ecosystem functioning. This study focuses on sediment data from Lake Plateliai from the last 130 years. This period is related to the fluctuations in the lake’s water level caused by the dam, the increase/decrease in primary productivity due to intensive agricultural development since the 1960s, and its decline in the 1990s.

The aim of this work was to assess how environmental factors have influenced the carbon cycle within the lake ecosystem and how these effects are recorded in the sediments. This includes the investigation of changes in sedimentation rates, carbon isotope distributions in the organic sediment fractions, fluctuations in diatom productivity, and shifts in community composition.

In this study, the content of organic matter, sedimentation rates, and diatom composition were analysed, along with a stable carbon isotope ratio and ^14^C specific activity measurements in two organic fractions of the lake sediment: alkali-soluble and alkali-insoluble. ^210^Pb and ^137^Cs dating methods were used to establish an independent chronology and to evaluate age variations in the lake sediments during the last century.

## Materials and methods

### Ethics statement

Fieldwork involving sediment core collection from Lake Plateliai did not require special permission, as the lake is state property and the participating institutions—the State Scientific Research Institute Nature Research Centre and the State Research Institute Center for Physical Sciences and Technology—are state-owned entities authorized to conduct such research.

### Site Description

**Fig 1:**
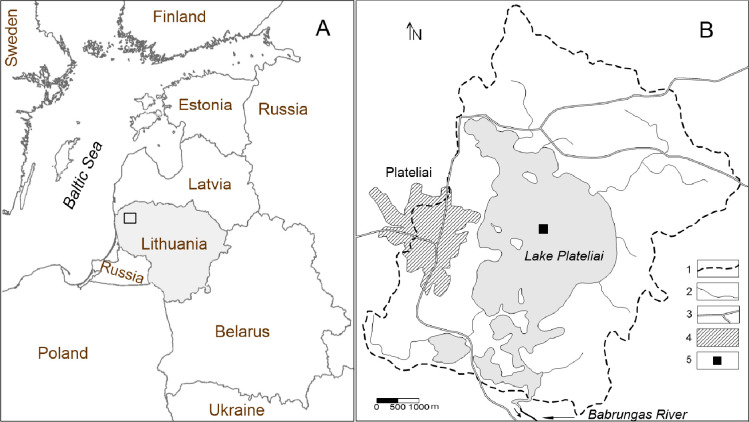
Location of the study area (A). Scheme of Lake Plateliai catchment **(B)**: 1 – catchment boundary; 2 – hydrographic network; 3 – main roads; 4 – main inhabitated area; 5 – sampling site. The scheme was made with Surfer software using data from geoportal.lt/map (accessed 11 June 2024).

Lake Plateliai is one of the largest lakes in Lithuania (N 56°2ʹ50ʺ, E 21°51ʹ23ʺ) and is situated within the watershed that separates the catchment areas of the Minija River (a tributary of the lower Nemunas), as well as the Venta and Bartuva Rivers, which flow directly into the Baltic Sea. The lake lies at an altitude of 146.48 meters above sea level (Fig 1). It covers an area of about 12 km² and reaches a depth of 49 m (maximum) with an average depth of 11.4 m [29]. The catchment area of Lake Plateliai is in the Samogitian plateau, a high plateau created by the last glaciation (13.3 ± 0.7 ^10^Be kyr BCE [30]), in a depression surrounded by various glacial forms. The bottom of this dimictic lake is a glacial depression with traces of subglacial erosion in the deepest parts of the lake [31].

The catchment area of Lake Plateliai itself is small (45.5 km², the length of the catchment area is 38 km), fairly symmetrical and has numerous small lakes with area of several hectares [29]. Lake Plateliai is in a mature stage, with a moderate nutrient status, as the inflowing waters are not heavily polluted. The lake is classified as mesotrophic, with an average chlorophyll-a concentration of 2.8–4.9 µg/l during the summer-autumn phytoplankton bloom [32,33]. The annual water exchange rate is up to 12%. Seven small streams flow into Lake Plateliai and only one outflow – the Babrungas River. Lake Plateliai was dammed on the Babrungas River for the purposes of the Babrungas water mill at the end of the 18th century. The water level of Lake Plateliai rose by approximately 1.5-2 m during this time, which led to severe erosion of the shoreline. In 1960, a new dam was built at the southern end of the lake, which was dammed during the construction of the hydroelectric power plant on the outflowing Babrungas River. This hydrotechnical activity significantly changed the water regime of Lake Plateliai: it increased the annual amplitude of water level fluctuations and maintained the water level in the lake for a long time. Between 1960 and 1978, the waters of Lake Plateliai were used to cool the missile launch pad of a nearby Soviet military base located 1.6 km away. This was possibly the cause of the fluctuating water levels of up to 0.8 metres during this period.

Contemporary climate variability is also observed in the time series of the water temperature of Lake Plateliai [34]. The mean water temperature (°C) of Lake Plateliai during the summer months in the surface water layer increased slowly in the periods 1964–1971, 1981–1990 and 1991–2000 and amounted to 14.6, 14.7 and 15.2 °C, respectively. In the bottom water layer, the temperature in the summer months has risen by 1.6 °C since the period 1976–1983 compared to the period 1984–1997.

Lake Plateliai and its surroundings have long been recognized as an ecologically valuable and protected area. The Plateliai Protected Landscape Area was established in 1960, and with the expansion of Lithuania’s protected area system, the Samogitia National Park was founded in 1991. Today, Lake Plateliai lies at the heart of the Samogitia National Park. The surrounding landscape is characterized by rolling hills, peat bogs, and numerous small lakes, creating diverse natural habitats that support a rich variety of flora and fauna.

For the present study, a 37 cm long sediment core was taken from the ice-covered lake on 22 February 2017 in the deepest part of the lake (depth 49.5 m, position 56^o^02’50.4’‘ N, 21^o^51’21.3’‘ E, Fig 1) using a Kajak gravity corer with an acrylic sample tube (inner diameter 52 mm). The core was cut into 1 cm slices *in situ* using a piston rod immediately after sampling. The samples were transported to the laboratory in containers and stored at −20˚C until analysis.

### Sample preparation and measurements

#### ^137^Cs, ^241^Am, ^210^Pb and ^214^Pb Measurements in Sediment Samples.

The measurements of ^210^Pb (at 46.5 keV), ^226^Ra (from ^214^Pb and ^214^Bi at 295, 352 and 609 keV), ^241^Am (at 59.5 keV) and ^137^Cs (at 661 keV) activity in lyophilized sediment samples were performed on a gamma-ray spectrometer with HPGe GWL-series detector (resolution 2.25 keV at 1.33 MeV) using 3 cm^3^ geometry sample vials fitting well geometry in a HPGe well-type detector as described in [23,35]. The detection limit at a counting time of 200,000 s was about 0.065 Bq for ^210^Pb, 0.021 Bq for ^226^Ra (from ^214^Pb), 0.006 Bq for ^241^Am and 0.014 Bq for ^137^Cs, and the measurement errors did not exceed 15%, 20%, 30% and 8% for ^210^Pb, ^214^Pb, ^241^Am and ^137^Cs, respectively. The uncertainties (±2s) in the activity of radionuclides in the samples were evaluated using the GammaVision-32 software. The quality of the gamma spectrometric measurements was ensured by regular analysis of control samples and normal precision was established in multiple comparisons.

To reconstruct the chronology of recent sediments from the activity of ^210^Pb in lake sediments, the constant rate of the ^210^Pb supply (CRS) model [36–38] was used with some modifications [24,39]. Since the total ^210^Pb activity in lake sediments consists of two components, the supported activity resulting from *in situ* decay of ^226^Ra in the sediments (in secular equilibrium with ^210^Pb), and the unsupported ^210^Pb arising from atmospheric fallout and used for dating, the unsupported ^210^Pb activity was derived by subtracting the ^214^Pb activity from the total ^210^Pb activity. The ^210^Pb excess was used for the sediment age calculation based on radioactive decay. The combination of ^210^Pb chronology with ^137^Cs and ^241^Am activity peaks from 1963 and ^137^Cs activity peak from 1986 was used to validate the age versus depth relationship of the lake sediment core.

#### Radiocarbon Dating.

The measurements of specific radiocarbon activity were carried out in two distinct organic fractions extracted from the lake sediment: one soluble in alkali and the other consisting of organic matter that is insoluble in alkali. We decided to use terms “alkali-soluble” and “alkali-insoluble” proposed by Markus Kleber and Johannes Lehmann [40] instead of “humic acids” and “humin”, since alkaline extraction cannot reliably distinguish between “humic substances” (materials that had undergone secondary synthesis in soils or “humification”) and “non-humic” substances.

Sediment samples underwent pretreatment using the acid-base-acid (ABA) method for the extraction of two fractions [24,41]. The sequential washes included a 1M HCl wash overnight, followed by a 0.2M NaOH wash for 1 hour (with the base solution being replaced until it remained colourless), and ended with another 1M HCl wash for 1 hour to obtain the alkali-insoluble fraction. The alkali-soluble fraction was subsequently extracted from the base solution by acidification. Prior to the radiocarbon measurements, the samples underwent a graphitization process using automated graphitization system (AGE-3, Ionplus AG). A single-stage accelerator mass spectrometer (SSAMS, NEC, USA) was used for radiocarbon measurements. The article [42] provides information on the typical parameters of the SSAMS system. The background of the measurements was estimated to be 0.25 pMC (percent of modern carbon) using phthalic anhydride (Alfa Aesar). The IAEA-C3 (129.41 pMC), IAEA-C9 (0.12-0.21 pMC), NIST-OXII (134.06 pMC) standards were used as reference materials. The accuracy of measurement the ^14^C/^12^C ratio was better than 0.3%. For the isotopic fractionation correction, the ratio of ^13^C to ^12^C was used.

#### ^13^C/^12^C, OC, TN, and OM Measurements in Sediments.

The content of organic carbon (OC) and total nitrogen (TN) in the sediments was determined using a Thermo Flash EA 1112 elemental analyser. The standard sulphanilamide (Merck, cat. #111799) was used for calibration. The long-term standard measurements were performed with a precision of <1% for N, and <1.5% for C. Carbonates were removed prior to analysis. The samples were acidified with 1M HCl, then rinsed in deionized water to a neutral pH and dried at 60 °C in a drying oven overnight.

Stable carbon isotope ratio measurements in alkali-soluble, and alkali-insoluble organic fractions were performed using a Thermo Flash EA 1112 elemental analyser connected to a Thermo Scientific Delta V Advantage isotope ratio mass spectrometer (IRMS).

All the results of the stable isotope ratio measurements were expressed relative to a standard using delta notation, with values reported in permil (‰):


$$\delta^{\text{1}\text{3}}\text{C}=\left(\frac{{\text{R}}_{\text{sample}}}{{\text{R}}_{\text{standrd}}}-\text{1}\right)\times {\text{1}\text{0}}^{\text{3}};$$

where R = ^13^C/^12^C in the sample or in the standard. The stable carbon isotope ratios were expressed in relation to the international reference standard Vienna Pee Dee Belmnite (V-PDB). IAEA600 and IAEA CO-8 were used to calibrate the laboratory standards for measurements of organic matter and carbonates, respectively. The long-term reference material measurements were performed with a precision of <0.1 ‰ for C in organic matter.

For the determination of OM (organic matter) content in sediments, the loss on ignition method was used [43]. Before combustion at 550^o^C for 5 h to determine the weight loss, the sediments were dried at 105^o^C for 12 hr. The organic matter accumulation rate was calculated from organic matter content data and the sediment mass accumulation rate (SMAR).

#### Sediment Particle Size Analysis.

The particle size analysis was carried out using the ANALYSETTE 22 MicroTec-plus laser particle sizer. Before measurement, each sediment sample was freeze-dried and ground. The measurements were carried out over the entire measurement range (0.1-2000 μm), with triplicate samples analysed to obtain mean values. The analysis yielded a volume-based particle size distribution expressed as percentages. The Udden-Wentworth grain-size scale was applied for sediment classification and description [44].

#### Diatom analysis.

The diatoms for the analysis were collected at 2 cm intervals along the sediment core. A total of 20 sediment samples were analysed. Sediment samples were treated with 30% hydrogen peroxide, following the methods described in [45,46]. Diatoms were mounted on coverslips using the Naphrax™ medium. At least 500 valves per slide were counted under a light microscope at 1000 × magnification. Diatom species identification was based on references [47–50] and classified according to their ecological preferences as described in [51]. Species names were verified using the AlgaeBase database taxonomy [52]. Only dominant diatom species (more than 5% of total abundance) were included in the analysis and diagram, as they best reflect the main environmental changes. A diagram of the distribution of taxa per sediment core was created using TILIA and TILIA-GRAPH software [53].

#### Magnetic susceptibility analysis.

The low-field magnetic susceptibility (κ) of the samples from Lake Plateliai was measured at the European Centre for Geological Education in Chęciny, Poland. The measurements were performed using the Multi-Function Kappabridge device (MFK1-FA, AGICO) with frequencies of 976 and 15,600 Hz, a sensitivity of 2 × 10^−8^ SI, and a magnetic field (H) of 200 A·m^−1^. The mass specific magnetic susceptibility (χ) was calculated by dividing κ by the mass of the sample [54,55].

## Results

### ^137^Cs and ^210^Pb Sediment dating and Sedimentation Rate Determination

The data on the activity concentration of ^210^Pb (total), ^214^Pb, and ^137^Cs in the sediment core versus depth in 1 cm resolution to 37 cm depth are given in S1 A Fig.

The specific activity of the total ^210^Pb exponentially decreased from 1070-1165 Bq/kg in the 0–4 cm depth interval of the sediment core to 66–70 Bq/kg at a depth of 35–37 cm, but with positive (1165 ± 64 Bq/kg at a depth of 3.5 cm, 985 ± 73 Bq/kg at a depth of 8.6 cm, 360 ± 37 Bq/kg at a depth of 18.8 cm) and negative (228 ± 68 Bq/kg at a depth of 15.7 cm) ^210^Pb deviations from exponential function. These small changes could be influenced by many factors, including the ^210^Pb atmospheric flux and the rate of its transport from the catchment; the water residence time; the fraction of radionuclides attached to settling particles and the mean settling velocity of particles; as well as various post-depositional transport processes [36]. Based on the conventional statistics (^214^Pb average value in sediment core plus 2 standard deviations), the maximal level of ^214^Pb in sediments from decaying of ^226^Ra *in situ* was evaluated to be 44 Bq/kg. In the studied core, at a depth of 35–37 cm, there is still an excess of ^210^Pb, ^210^Pb_ex_, relative to ^214^Pb up to 27–56 Bq/kg.

The ^210^Pb excess was used for the sediment age calculation (S1 B Fig.) based on radioactive decay using CRS model. The profile of ^210^Pb_ex_ in the sediment core to a depth of 37 cm tended to decrease exponentially with the mass depth, the product of the wet thickness of the sediment slice and the dry bulk density d_m_ (^210^Pb_ex_ = 1120 × exp(−1.056 × d_m_); R^2^ = 0.95). This resulted a rather constant ^210^Pb_ex_ flux and steady-state sedimentation with an average sediment mass accumulation rate (SMAR) of 0.029 g/cm^2^/y. This average SMAR value was used to calculate the sediment age and partial SMAR values for the studied core up to the depth of 37 cm (S2 A Fig.).

The sediment age of the lowest part of the core can be dated to 1885 ± 17 CE. The partial SMAR values for the past 130 years have been in the range of 0.052 ± 0.022 to 0.019 ± 0.008 g/cm^2^/y with an average SMAR value of 0.029 g/cm^2^/y. The average of 2σ uncertainties for the SMAR was 18%. The average SMAR value corresponded to the linear sedimentation of wet matter equal to 0.38 cm/y.

The data on ^137^Cs and ^241^Am (decay product of ^241^Pu) fallouts from the atmosphere due to nuclear weapons testing in the atmosphere, as independent chronostratigraphic markers well confirmed chronology based on ^210^Pb. Both peaks, ^137^Cs and ^241^Am, were found in the same sample (depth 20 cm) and dated to 1963 ± 2.5 CE (S2 B Fig.).

The data on the fallout of ^137^Cs from the atmosphere due to the accident at the Chernobyl Nuclear Power Plant (NPP) did not contradict the ^210^Pb chronology either. A pronounced peak of ^137^Cs (324 ± 14 Bq/kg) from the Chernobyl NPP accident was found at a depth of 10 cm, which was dated to 1992 ± 1.2 CE according to the ^210^Pb chronology. The time lag of ~5 years between Chernobyl NPP accident in 1986 and ^137^Cs peak attribution in ^210^Pb chronology to 1992 could be related to the delay in the transfer of ^137^Cs from the catchment area and various post-depositional transport processes, including the diffusion of ^137^Cs in sediments and their bioturbation.

### Carbon isotope measurements in organic sediment fractions

**Fig 2:**
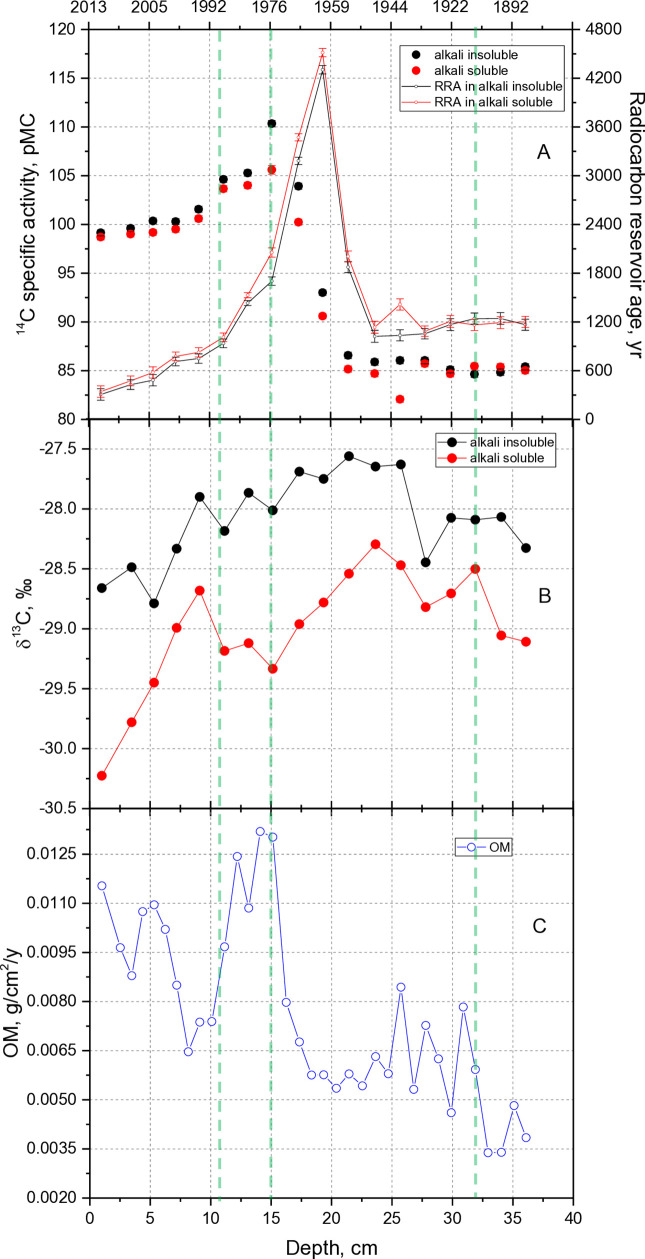
Radiocarbon specific activity. **(A)**, δ**^13^C ratio values (B) in both sediment organic fractions, as well as sedimentary organic matter (OM) accumulation rate based on ^210^Pb chronology** **(C)**. Plotted the reservoir age (RA) data (A) were established in both sediment organic fractions. Green lines separate the time periods examined.

The ^14^C specific activity in the alkali-insoluble organic fraction ranged from 84.6- to 110.3 pMC, whereas ^14^C specific activity in the alkali-soluble organic sediment fraction fluctuated in the range of 82.0 to 105.6 pMC over the 128-year period (1885–2013) studied (Fig 2A). Both organic fractions showed the same values for specific radiocarbon activity within ±1 pMC, except for the depth interval of 26–15 cm (1941–1976), when the specific radiocarbon activity in the alkali-soluble fraction was 1.5-4.7 pMC higher than the values in the alkali-soluble fraction. As can be seen in the ^137^Cs profile (S1 A Fig.), the peak activity value was observed at 19 cm, while in the ^14^C activity profile it can be seen at 16 cm, i.e., with an offset of 10 years.

The stable carbon isotopic composition of both sediment organic fractions is shown in Fig 2B. The δ^13^C values in the alkali-insoluble organic fraction varied between −28.8‰ and −27.6‰ and were up to 1.5‰ more enriched in ^13^C than in the alkali-soluble organic sediment fraction, which varied between −30.2‰ and −28.5‰. The lowest δ^13^C values in both fractions were observed in top 5 cm sediment layers (period 2013−2005).

### Sediment Characteristics

The grain-size analysis revealed that the sediments of Lake Plateliai consist of silt, sand, and a very small (2–5%) admixture of the clay (S3 Fig.). The estimated proportions of sediment particle sizes indicate changes in the sedimentation regime within the lake. The sediments in the core consist of 50–80% silt-sized particles from the lowest layer to 11 cm below the surface. An abrupt decrease in silt and a corresponding increase in the sand fraction were observed in the interval from 16 to 17 cm in the upper part of the core. The upper layer (0–11 cm) is characterized by an increasing proportion of sand, reaching up to 50%. A composition of homogenous silt (with up to 30% very coarse-grained silt) and sand (with up to 37% very fine-grained sand) was also observed.

C/N values showed a decreasing trend from 10.4 to 8.2 throughout the study period, which could be an indication of decreasing allochthonous matter content in the sediments, an increase in primary producers, shifts in their community composition, or all three (S4 Fig.).

Organic matter (OM) accumulation rate values (Fig 2C) ranged from 0.0032 g/cm^2^/y to 0.0113 g/cm^2^/y exhibiting an increasing trend with maximum values of 0.0084 g/cm^2^/y, 0.0132 g/cm^2^/y, 0.0115 g/cm^2^/y at the depth of 33–25 cm (1904–1945), at 16–10 cm (1973–1992), and 6–0 cm (2002–2013), respectively.

The mass specific magnetic susceptibility values varied from 250 and to 1700 10^-9^m^3^/kg (S5 Fig.). Until 1922 (30 cm depth), the mass specific magnetic susceptibility varied within 250−300 × 10^-9^m^3^/kg. In the interwar period (in the depth interval of 30−24 cm), a clear increase in pollution was observed, which was expressed in higher values of the mass-specific magnetic susceptibility (300−500 × 10^−9^ m^3^/kg). Three peaks of values of 880 **×** 10^-9^m^3^/kg, 1020 × 10^-9^m^3^/kg, and 1650 × 10^-9^m^3^/kg were observed in 1970, 1985, and 2011 (at depths of 17 cm, 12 cm, and 2 cm, respectively).

In the case of Lake Plateliai, an increase in mass specific magnetic susceptibility was observed to coincided with the accumulation of thicker sediment layers at the bottom of the lake. It took 60–80 years to accumulate 10 cm of bottom sediments; during the period 1945–1990, the 10 cm sedimentation cycle required approximately 30 years; after 1990, it took 20 years only.

### Sedimentary Diatoms

A rich accumulation of diatoms with over 180 species, varieties and forms was found in the sediments of Lake Plateliai. The analysis of the diatoms showed that short-term changes in the environmental conditions in the lake have a noticeable effect on the diatom populations.

**Fig 3:**
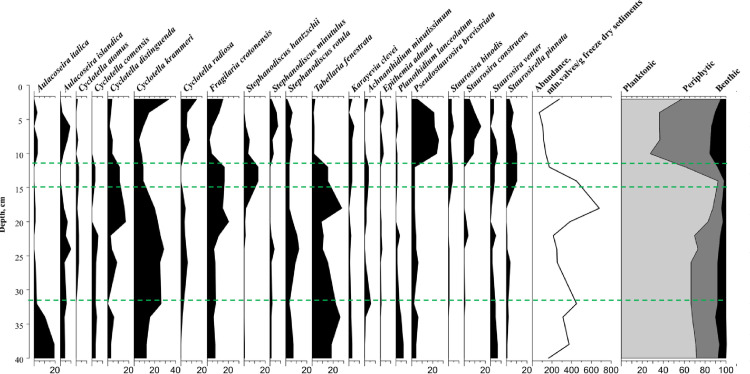
Vertical distribution of relative abundance of diatom taxa, diatom groups according to ecological preference, and total amount of the valves in the sediment core of the Lake Plateliai. Green lines separate the time periods examined.

In the lower and middle layers of the sediment core (40–21 cm), diatom abundance varied from 162.32 × 10^6^ to 449.37 × 10^6^ valves g^-1^ of sediment dry weight (Fig 3). A significant increase in abundance, reaching 682.56 × 10^6^ valves g^-1^, was observed at the 18–19 cm layer, followed by a sharp decline in the upper layers (12–2 cm), where valve abundance decreased by 5–9 times, marking the lowest levels within the core.

The Tillia analysis revealed diatom composition changes. Overall, a dominance of planktonic species was assessed, including *Aulacoseira italica*, *Aulacoseira islandica*, *Fragilaria crotonensis*, *Tabellaria fenestrata*, and species from the *Cyclotella* genus, primarily *C. krammeri* and *C. distinguenda*, with up to 60% relative abundance. In the upper layer, however, the concentration of planktonic diatoms decreased drastically, and the assemblage shifted to a periphytic community dominated by fragilarioid taxa, particularly *Pseudostaurosira brevistriata*, *Staurosira construens*, *Staurosirella venter*, and *Staurosirella pinnata*.

## Discussion

Based on CONISS cluster analysis (S7 Fig.) of SMAR/sediment organic fraction accumulation rate (Figs S2A and 2C) and the grain size distribution data (S3 Fig.), four research periods were divided: 1) the period of 1885−1910 (depth of 36−32 cm); 2) the period of 1910−1976 (depth of 32−15 cm); 3) 1976−1988 (depth of 15−11 cm), the period of the highest organic matter accumulation rate, but also showing the trend of highest decline in diatom abundance, especially of planktonic taxa; 4) the period after 1988 (depth of 11−0 cm) with the lowest total diatom abundance and the highest periphytic taxa, but after 1998 showing increasing trend in OM accumulation rate, which had decreased due to the collapse of agriculture in the 1990s.

### The period of 1885–1910

Radiocarbon concentrations remained stable between 1885 and 1910 (Fig 2A), and δ¹³C values in the alkali-soluble fraction also showed little variation during this interval (Fig 2B). However, between 1904 and 1910 (33–32 cm), the δ¹³C value of the alkali-soluble fraction increased by 0.6‰ to −28.5‰. This increase coincided with a significant shift in the diatom community, suggesting a possible trend of nutrient enrichment in the lake ecosystem. The abundance of *A. italica*, a species often associated with oligotrophic conditions [56], declined sharply; while *C. krammeri*, *C. distinguenda*, and *T. fenestrata* became more prevalent (Fig 3). The increased presence of *T. fenestrata* in the water column may serve as a potential indicator of nutrient input [51,57]. This ecological shift also coincided with a two-fold increase in organic matter accumulation rate and a decrease in C/N values (S4 Fig.) from 10.4 in 1885 to 9.5 in 1910.

In contrast, mass-specific magnetic susceptibility values did not exhibit abrupt changes and varied only within 250–333 × 10 ⁻ ⁹ m^3^/kg throughout 1885–1910 (S5 Fig.). Magnetic susceptibility can be treated as a parameter indicating the degree of sediment contamination (e.g., enrichment by trace elements) due to human economic activity, or as an indicator of erosion processes occurring in the catchment area. An increase in the value of this parameter may indicate an increase in anthropogenic pressure and transformation of the natural environment, while a decrease in this parameter may be the effect of the acceleration of erosion processes in the catchment area resulting from increased sediment supply to the lake [54,55,58,59].

No data on water-level fluctuations are available for 1885–1910, which limits the ability to evaluate hydrological influences. Nevertheless, the stable radiocarbon concentrations, together with the decrease in C/N values, changes in SMAR/OM accumulation rates (Figs S4, 2C, S2A), and the shift in δ¹^3^C of the alkali-soluble fraction, indicate that the observed changes between 1904 and 1910 were more likely related to transformations within the phytoplankton community and internal carbon cycling processes, rather than to changes in the relative contributions of autochthonous and allochthonous organic matter in the sediments.

### The period of 1910–1976

During the period 1910−1922, the OM accumulation rate changed, and the clay particles concentration decreased by 10%. However, the distribution of carbon isotopes in both organic sediment fractions during this period remained unchanged. At a depth of 28 cm (in 1932), the ^14^C specific activity increased by 1 pMC in both organic sediment fractions. This increase in radiocarbon concentrations was not related to the atmospheric radiocarbon specific activity during the first half of the 20th century, as atmospheric ¹⁴C gradually decreased by about 1.5 pMC between 1905 and 1950 (OxCal v4.2.4 [60,61]). Over the same period, the δ^13^C value in the alkali-insoluble sediment fraction decreased by slightly (0.4 ‰) to −28.5 ‰.

Diatom data do not support a major reorganization of aquatic vegetation associated with these isotopic shifts. Between 1910 and 1931 (at a depth of 32−28 cm), community composition showed no significant changes compared to the depth interval 32−33 cm. Only a slight decrease in *T. fenestrata* and a decrease in the total diatom abundance were observed, while the differences among the ecological groups of diatoms remained insignificant. The mass specific magnetic susceptibility values (S5 Fig.) gradually increased by 100 × 10^-9^m^3^/kg (from 300 to 400 10^-9^m^3^/kg**)** between 1910−1941 (32−26 cm), suggesting modification of sediment sources or catchment conditions.

A more pronounced shift occurred between 1941 and 1945 (26–25 cm), when mass-specific magnetic susceptibility increased by an additional ~100 × 10 ⁻ ⁹ m^3^ kg ⁻ ¹ (S5 Fig.), while the organic matter sedimentation rate decreased by 25% (Fig 2C). Minor changes in the diatom assemblage were detected, with a slight decline in total diatom abundance. A decrease in *T. fenestrata* and *C. distinguenda* was observed, while *Stephanodiscus rotula*, an indicator of eutrophic conditions, began to increase (Fig 3). The ^14^C specific activity value in the alkali-soluble sediment organic fraction decreased from 85.72 ± 0.33 pMC (at a depth of 28 cm) to 82.07 ± 0.31 pMC (at a depth of 26 cm), then increased to 85 ± 0.3 pMC at depth of 24 cm (in 1949) and remained stable until the ‘bomb peak’, when the concentration of ^14^C in the atmosphere doubled due to nuclear weapons testing (Fig 2A). In contrast, the ^14^C concentration in the alkali insoluble sediment organic fraction remained the same (of 86.14 ± 0.29 pMC) throughout 1932−1960 (28−20 cm) and began to increase only during the ‘bomb peak’ period. The δ^13^C value of this fraction (Fig 2B) increased by 0.8 ‰ up to −27.6 ‰ and varied within 0.2 until 1970 (at a depth of 17 cm).

There are no data on the events in the vicinity of Lake Plateliai in 1922−1932 (at a depth of 30−28 cm) and the period of the Second World War. However, the sedimentary changes observed during these periods (Figs. 2A, 2B, S4, and S5) are most likely related to two separate events, possibly caused by changes in the lake water level and the influx of allochthonous matter. These processes are expressed through changes in ¹⁴C concentrations in both organic fractions and, during the second event, by diatom-inferred increases in nutrient availability.

The slight increase in ^14^C specific activity in both organic sediment fractions at the depth 22 cm (corresponding to 1954) by 0.56 ± 0.32 pMC could be attributed to the beginning of nuclear weapons testing (Fig 2A). The difference in radiocarbon specific activity between the organic fractions of the sediments remained at 1.29 ± 0.45 pMC. The mass specific magnetic susceptibility values (S5 Fig) during the period of 1953−1956 (23−21 cm) increased by 210 × 10^-9^m^3^/kg (from 500 to 710 × 10^-9^m^3^/kg) indicating alteration of the environment. In 1953, diatom abundance began to increase, although this growth was not reflected in the organic matter accumulation rate. Significant shifts in the diatom community occurred: *C. krammeri* and *C. comensis*, which prefer oligotrophic-mesotrophic waters, and *S. rotula*, characteristic of mesotrophic-eutrophic lakes, began to decline. In contrast, the abundance of *F. crotonensis* and *T. fenestrata* increased significantly indicating a transition toward more eutrophic conditions, likely driven by rising nutrient concentrations (Fig 3).

Between 1960 and 1976, the carbon cycle of this lake ecosystem was primarily affected by three main environmental factors. First, the intensification of agricultural development in the Soviet Union, which began in the 1960s, led to increased runoff of mineral fertilizers into rivers and lakes. Second, in 1960, the installation of a flow regulation sluice in the southern part of the lake and the damming of the Babrungas River led to the creation of an artificial pond, which has since been used to maintain the operation of the Gondinga hydroelectric power plant (850 kW). According to witness accounts, the water level of the lake was artificially raised by several meters, maintained at this level for two years, and subsequently lowered to its normal level when the hydroelectric plant became operational in late 1962. Since then, the water level has been subject to artificial regulation based on the operational needs of both the power plant and a nearby Soviet missile launch base. The lake’s water was used as a cooling pond for the missile base’s launch systems. Third, the increase in nuclear bomb testing between 1950 and 1963 resulted in a doubling of atmospheric ¹⁴C concentrations, as reflected in the rising trend of specific ¹⁴C activity values in both fractions from 1960 to 1976 (at a depth of 20−15 cm, Fig 2A). Additionally, during this period, an increasing disparity in specific ¹⁴C activity values between the two fractions was observed, with the difference expanding from 2.4 to 4.7 pMC (the difference in RA would be 211.4 ± 51.7 y and 352.7 ± 57.4 y, respectively). Simultaneously, mass-specific magnetic susceptibility increased from 600 to 850 × 10 ⁻ ⁹ m^3^/kg (S5 Fig). Typically, the alkali-soluble (humic acid) fraction exhibits a higher ¹⁴C concentration than the alkali-insoluble (humin) fraction [2,62–65]. However, in this ecosystem, the alkali-soluble fraction displayed a lower radiocarbon concentration, indicating potential deviations from expected carbon cycling dynamics.

After the 1960s, the highest absolute abundance of diatoms, especially planktonic species (Fig 3), and the dominance of species thriving in mesotrophic-eutrophic conditions such as *Stephanodiscus hantzschii*, *F. crotonensis*, *T. fenestrata* were observed. *S. hantzschii*, compared with *Fragilaria* and *Tabellaria* species, thrives in more eutrophic waters with elevated phosphorus levels, often indicating periods of increased nutrient loading and shifts in the trophic state of the lake [66,67]. In addition, an increasing trend was observed in the organic matter accumulation rate, as well as a decreasing trend of δ^13^C values in the alkali-soluble fraction (the highest value of 0.00797 g/cm^2^/year and the lowest value of −29.3‰ were observed in 1976–1980 at 15–14 cm, Figs 2C and 2B, respectively). Notably, these changes did not coincide with the period of highest agricultural activity and fertilizer use in Lithuania, which occurred in 1989–1990. C/N values remained stable during 1960–1976 (S4 Fig.). Considering the data on water level changes in the lake during 1963–1976, it can be assumed that the increase in organic matter accumulation rate and the difference in ^14^C concentrations between the two organic sediment fractions were not caused only by the intensification of photosynthesis. It is the input of organic material of allochthonous origin into the lake ecosystem, such as the 1941 event (at a depth of 26 cm), which also “compensated” for the decrease in C/N ratio with increasing photosynthetic intensity and altered the redistribution of ^14^C between the fractions (Fig 2A).

### The period of 1976–1988

In 1976, the lake’s water level dropped sharply by 75 cm (S6 Fig), returning to its former level in 1977. This change in water level probably resulted in a maximum difference of 4.74 ± 0.45 pMC ^14^C specific activity value between the two organic fractions (Fig 2A). Between 1980 and 1992, water-level fluctuations did not exceed 30 cm (Fig. S6), and the difference in ¹⁴C specific activity between the fractions decreased by 1.1 ± 0.4 pMC (Fig 2A). Over the same interval, the ¹⁴C specific activity of the alkali-insoluble fraction declined by 3.1 ± 0.4 pMC relative to 1976, reaching 105.3 ± 0.3 pMC in 1982 (Fig 2A). This decrease in radiocarbon concentration was, however, most likely related to the decrease in atmospheric ^14^CO_2_ concentration after the ’bomb peak’ (in the lake ecosystem, this peak was observed only in 1976, 13 years after the maximum ^14^C concentration in the atmosphere).

During the period 1976−1985, the highest values of OM accumulation rate (0.011 to 0.013 g/cm^2^/yr, Fig 2C) were observed, although the total diatom abundance decreased by a factor of 2.6 during this period (Fig 3), with a steep decrease in the relative concentrations of the planktonic diatom species, and an increase of the concentrations of the periphytic and benthic diatom species. Changes in the diatom community did not affect the distribution of stable isotopes in the sediment organic fractions. The δ^13^C value in the alkaline soluble fraction, which reached its minimum value of −29.3‰ since 1885, remained constant throughout the period 1976−1988. The ^13^C/^12^C ratio in the alkaline insoluble fraction, which decreased slightly (0.3‰) in 1976, remained constant thereafter (−28 ± 0.2‰) until 1995. The distribution of stable isotopes in sediment organic fractions was not affected by coastal erosion (seen increase in the relative concentration of sand particles from 15 to 41%, S3 Fig) due to the construction of holiday resorts in the 1980s, which was the reason of an increase by 260 × 10^-9^m^3^/kg in the mass specific magnetic susceptibility values observed in 1985 (S5 Fig). However, it is likely that the high OM accumulation rate values observed after 1976 are not related to the possible input of allochthonous organic matter into the lake ecosystem due to shore erosion. There were no differences in radiocarbon concentrations in the two organic fractions due to the input of allochthonous organic matter as previously observed, and the values of stable carbon isotopes in both organic fractions remained constant. The high OM accumulation rate values are most likely related to the dominance of phytoplankton species other than diatoms in the ecosystem (the reduced δ^13^C values also indicate that the dominance of macrophytes is unlikely, as they are generally more enriched in the ^13^C isotope and have higher C/N values [68]).

### The period after 1988

As noted previously, the maximum intensity of fertilizer application in Lithuanian agriculture occurred in 1989–1990. Paradoxically, the associated increase in nutrient inputs coincided with a decline in organic matter (OM) accumulation (Fig 2C), which reached its minimum in 1998. Following the 1990s, collective farms were dismantled and former holiday resorts ceased operation, while the lake area was incorporated into the Samogitia National Park. However, by the late 1990s, intensive construction of private housing began around the lake. Although wastewater treatment facilities were introduced in 1995, their performance remains suboptimal. Nitrogen limitation, combined with elevated phosphorus concentrations resulting from wastewater discharge into the lake ecosystem, has led to a shift in the phytoplankton community, favoring the dominance of diazotrophic cyanobacteria, as reported in previous studies [32,69,70]. As a result, an increase in organic matter accumulation rate, decline in C/N values (S4 Fig.), and a pronounced decreasing trend in the δ¹^3^C values of the alkali-soluble fraction in the upper sediment layers (from −28.6 ‰ to −30.25 ‰, Fig 2B) were observed.

Notably, diatom concentrations between 1988 and 2011 (at a depth of 11−3 cm) were the lowest recorded within the entire 130-year study period. Changes in diatom communities reflect variations in water level and shifts towards mesotrophic – eutrophic conditions. The abundance of planktonic species declined, while the proportion of benthic diatoms, particularly periphytic taxa, increased significantly, reaching up to 75%. Species associated with nutrient enrichment, such as *F. crotonensis*, *P. brevistriata*, *Stephanodiscus minutulus*, *S. rotula*, and *C. radiosa* became dominant (Fig 3). These taxa serve as indicators of ecological change driven by increased nutrient input. In particular, *F. crotonensis* is considered a marker of reactive nitrogen levels [71] and suggests that concentrations exceeded the critical threshold after 2009.

The decreasing trend in radiocarbon concentrations in both organic fractions within the upper 13 cm of the core is most likely associated with the decline in atmospheric ¹⁴C. Although no lake-level measurements are available for 1992–2013, no substantial changes in water level were reported for this period. A possible exception is 2002, when the difference in radiocarbon concentrations between the two fractions increased to 1.2 pMC and the δ¹^3^C value in the alkali-insoluble organic fraction decreased slightly by 0.5‰ to −28.8‰ (Figs 2A and 2B). The twofold increase in magnetic susceptibility (S5 Fig.) in sediments deposited around 2010 may be attributed to the exceptionally high precipitation levels recorded that year in Lithuania and across Central Europe. This period ranks among the wettest in the history of hydrometeorological observations [72,73].

After 2011 (at a depth of 3 cm), the ^14^C specific activity values in both sediment fractions equalized within the error limits, but the specific activity values did not return to the ‘pre-bomb peak’ levels. The age of the organic sediment fractions in the lake ecosystem has changed by 872.4 ± 80.2 y compared to the pre-bombing value (Fig 2A). In the very upper layers, it is seen that radiocarbon concentrations in both fractions are still decreasing. In 2013, an unregulated sill was installed at the Babrungas River outflow, maintaining stable lake’s water level at approximately 146.48 m. From then on, changes in the carbon cycle in the lake will be more dependent on processes in the lake itself. Changes in the phytoplankton community, in particular the establishment of diazotrophic cyanobacteria, are certainly changing the rate of CO_2_ exchange between the atmosphere and the lake ecosystem. It will take several decades to determine what, if any, ‘steady state’ freshwater reservoir value will be present in the sediment organic fraction.

## Conclusions

This study demonstrates how environmental factors have influenced the lake’s carbon cycle and how these changes have been preserved in sediments over the past 130 years. The radiocarbon reservoir age has decreased by approximately 872.4 ± 80.2 years, with a declining trend in ¹⁴C concentration in the upper layers. The specific ¹⁴C activity values in both organic sediment fractions remained stable during the last decade and between 1885 and 1932. However, water level fluctuations between 1963 and 1976, along with unidentified events from 1932 to 1941, facilitated the influx of allochthonous material into the lake ecosystem, leading to a relative decrease in ¹⁴C concentration in the alkali-soluble fraction compared to the alkali-insoluble fraction. The δ^13^C values in the alkali soluble sediment organic fraction showed a tendency to decrease during the periods of increased nutrient input, coinciding with shifts from oligotrophic to mesotrophic or even eutrophic conditions. Since the 1990s, a decline in planktonic diatoms and an increase in benthic species have been observed, indicate an ongoing ecological imbalance within the system.

## Supporting information

S1 Fig Lake Plateliai sediment records: A) ^137^Cs and ^210^ Pb profiles, error bars represent uncertainties based on the propagation of 2 σ counting errors; B) model ages from ^210^Pb profile. (TIF)

S2 Fig Lake Plateliai sediment records: (A) sediment mass accumulation rates (SMAR) based on ^210^Pb chronology; (B) ^137^Cs and ^241^Am profiles. (TIF)

S3 Fig Sediment particle size distribution in the core of Lake Plateliai. (TIF)

S4 Fig C/N ratios in sedimentary organic matter from Lake Plateliai. (TIFF)

S5 Fig The mass specific magnetic susceptibility (χ) versus the depth of the sediments. (TIF)

S6 Fig The variations in water level. (TIF)

S7 Fig Dendrogram obtained from SMAR/sediment organic fraction accumulation rate and the grain size distribution data using CONISS. (TIF)
