# Supplementary figures and images for: Environmental changes and their effects on carbon isotope distribution in Lake Plateliai over the past 130 years: insights from sediment organic fractions and diatom assemblages
Source: PLoS One. 2026 Mar 4;21(3):e0343824. doi: 10.1371/journal.pone.0343824 (PMC12959667; doi:10.1371/journal.pone.0343824)

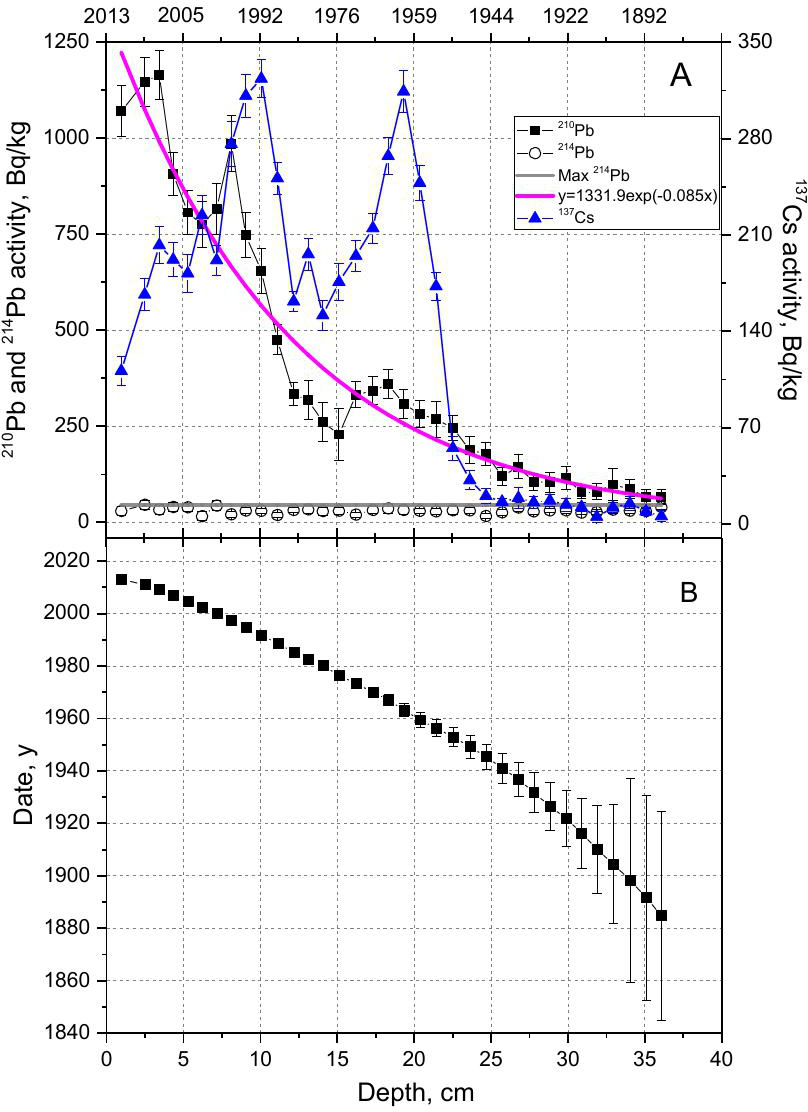

Supplement: S1 Fig — (TIF) [file pone.0343824.s001.tif]

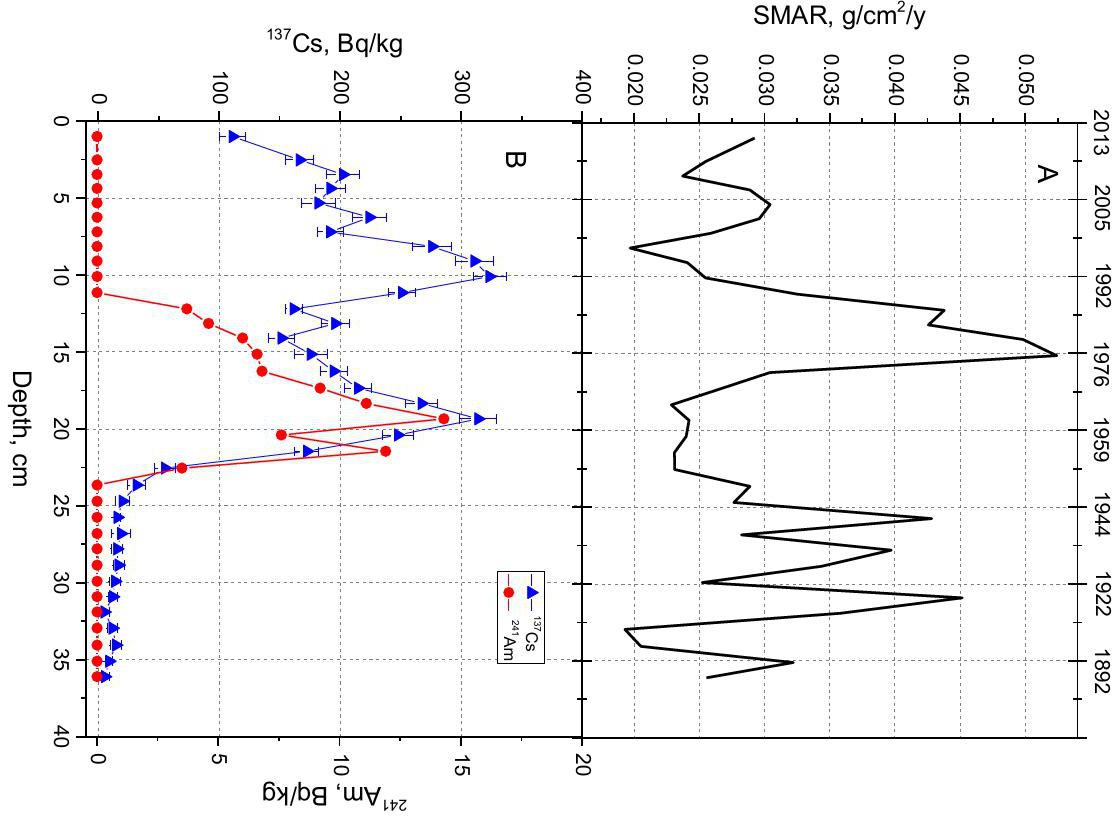

Supplement: S2 Fig — (TIF) [file pone.0343824.s002.tif]

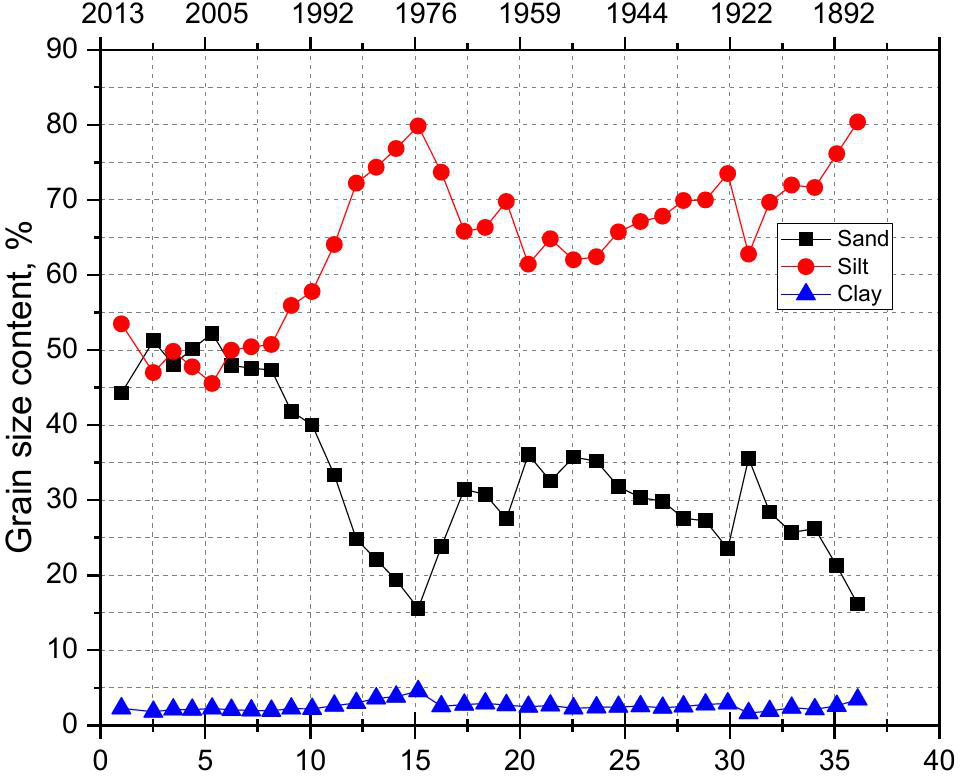

Supplement: S3 Fig — (TIF) [file pone.0343824.s003.tif]

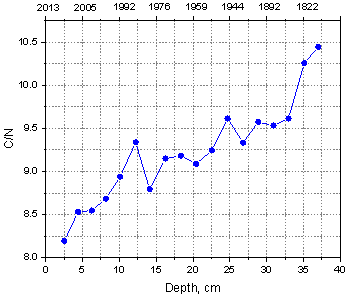

Supplement: S4 Fig — (TIFF) [file pone.0343824.s004.tiff]

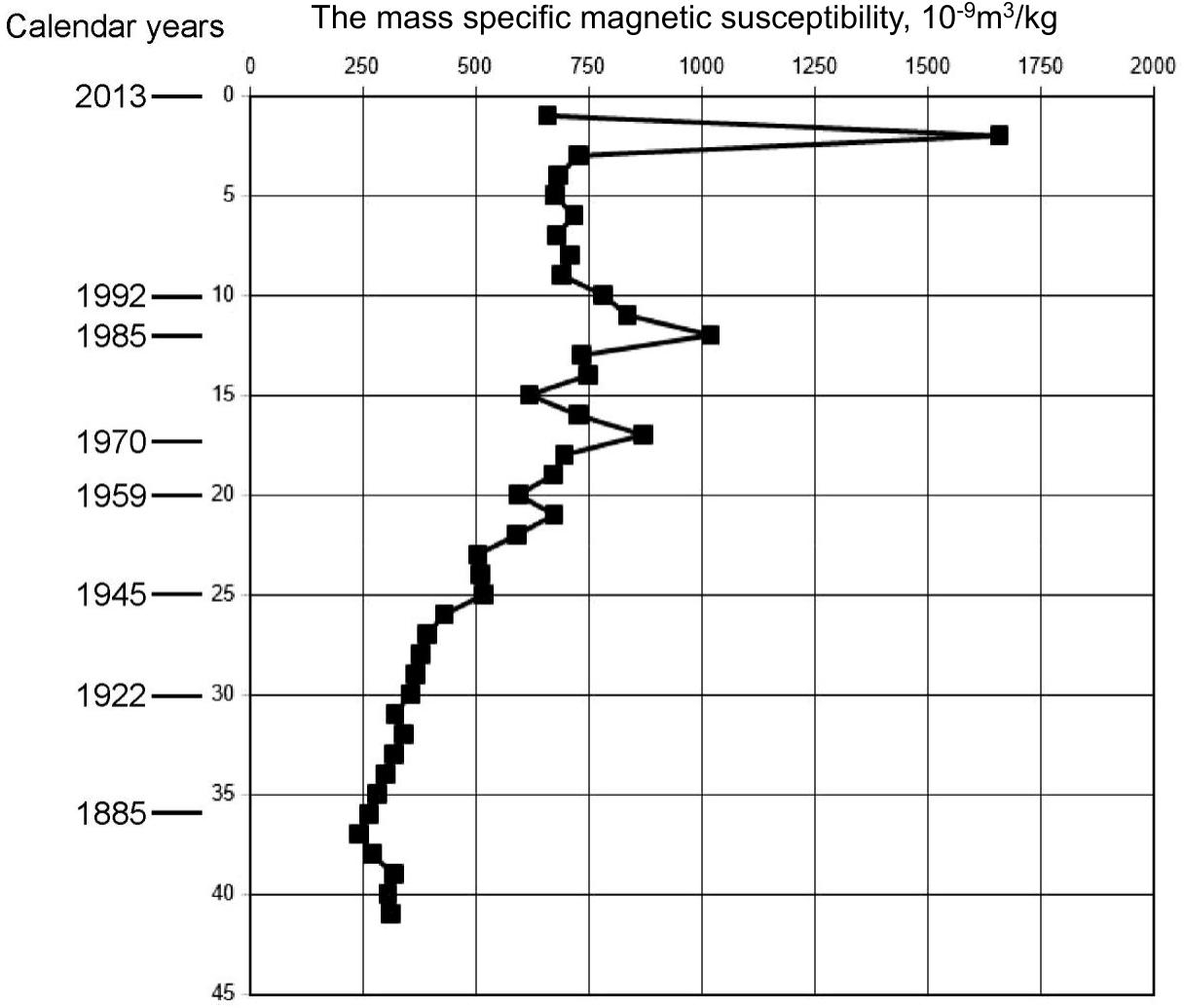

Supplement: S5 Fig — (TIF) [file pone.0343824.s005.tif]

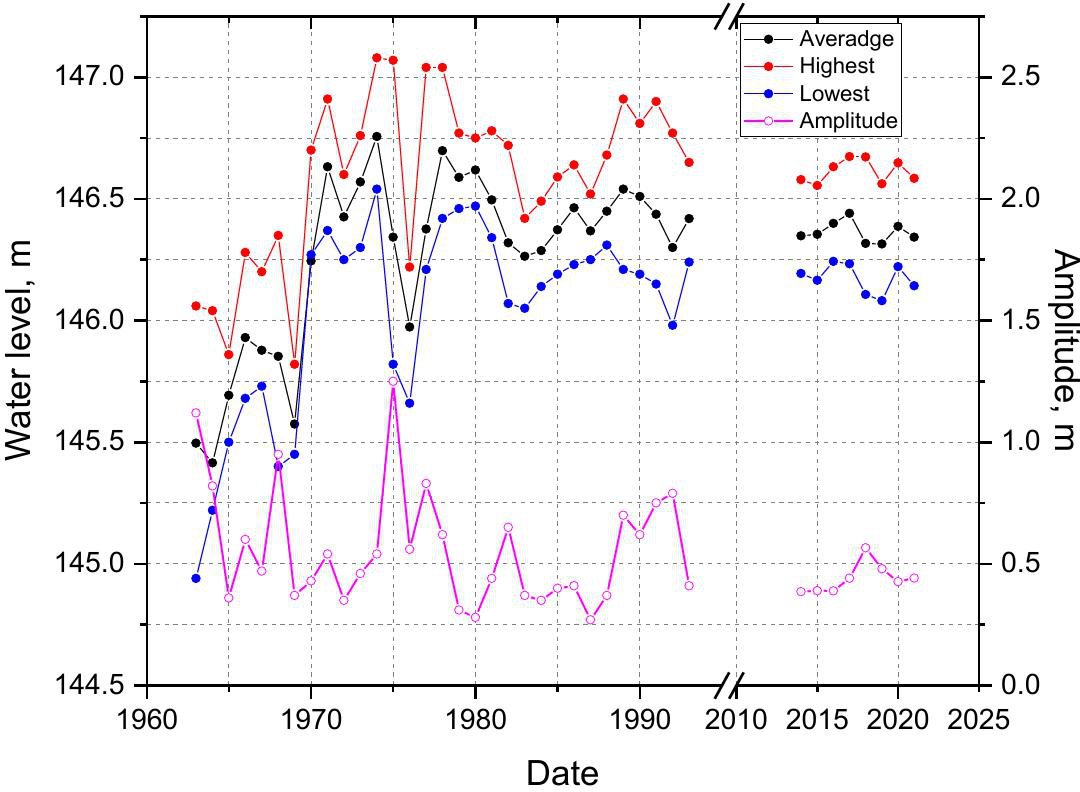

Supplement: S6 Fig — (TIF) [file pone.0343824.s006.tif]

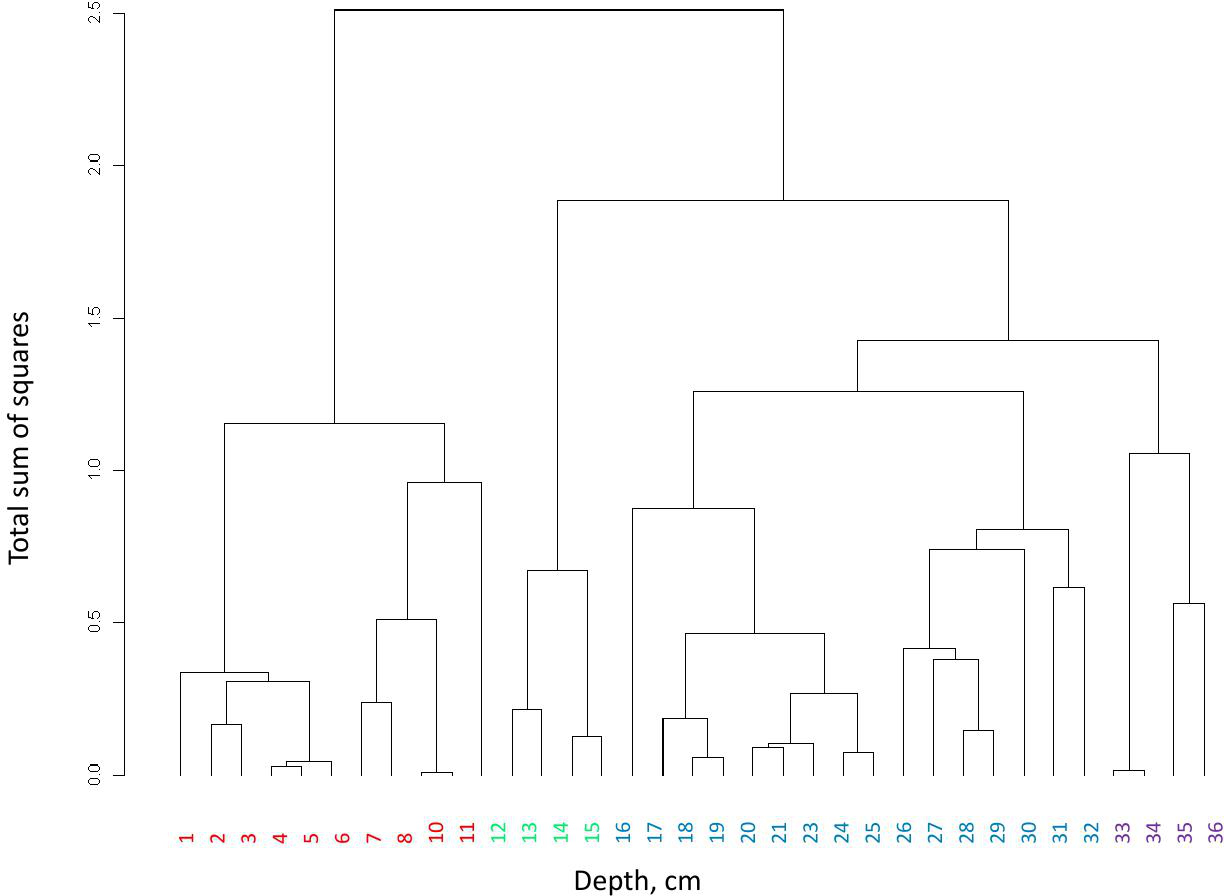

Supplement: S7 Fig — (TIF) [file pone.0343824.s007.tif]
